# Molecular Assessment of Healthy Pathological Articular Cartilages in Physically Active People: A Scoping Review

**DOI:** 10.3390/ijms24043662

**Published:** 2023-02-11

**Authors:** Luca Petrigna, Bruno Trovato, Federico Roggio, Alessandro Castorina, Giuseppe Musumeci

**Affiliations:** 1 Department of Biomedical and Biotechnological Sciences, Section of Anatomy, Histology, and Movement Science, School of Medicine, University of Catania, Via S. Sofia No. 97, 95123 Catania, Italy; 2 Sport and Exercise Sciences Research Unit, Department of Psychology, Educational Science and Human Movement, University of Palermo, Via Giovanni Pascoli 6, 90144 Palermo, Italy; 3 Laboratory of Cellular and Molecular Neuroscience (LCMN), School of Life Sciences, Faculty of Science, University of Technology Sydney, Sydney, NSW 2007, Australia; 4 Research Center on Motor Activities (CRAM), University of Catania, Via S. Sofia No. 97, 95123 Catania, Italy; 5 Department of Biology, Sbarro Institute for Cancer Research and Molecular Medicine, College of Science and Technology, Temple University, Philadelphia, PA 19122, USA

**Keywords:** biomarkers, physical fitness, osteoarthritis, standardization, articular cartilage

## Abstract

Physiological aging triggers a cascade of negative effects on the human body and the human joint is only one of the several compartments affected by this irreversible and natural process. Osteoarthritis and cartilage degeneration can cause pain and disability; therefore, identifying the molecular processes underlying these phenomena and the biomarkers produced during physical activity is of critical importance. In the present review, the main goal was to identify and discuss the articular cartilage biomarkers analyzed in studies in which physical or sports activities were adopted and eventually to propose a standard operating procedure for the assessment. Articles collected from Pubmed, Web of Science, and Scopus were scrutinized to detect reliable cartilage biomarkers. The principal articular cartilage biomarkers detected in these studies were cartilage oligomeric matrix protein, matrix metalloproteinases, interleukins, and carboxy-terminal telopeptide. The articular cartilage biomarkers identified in this scoping review may aid in a better comprehension of where research on the topic is heading and offer a viable instrument for streamlining investigations on cartilage biomarker discovery.

## 1. Introduction

Aging is an irreversible and inevitable process that causes the progressive deterioration of several structures of the human body. One of the most affected tissues during aging is the cartilage; it is often the initial site of degenerative processes, a phenomenon most likely due to its reduced self-healing potential, particularly after repeated injuries [1]. Aging impairs cartilage tissue cell turnover, causing ongoing chondrocyte loss, altered functionality [2], reduced metabolic response, functional changes in the matrix, and changes in synovial tissue composition [3]. Cartilages represent the fundamental components of articular joints, and are functional units essential for human movement, allowing the transmission of the mechanical load between different mobile parts of the body with minimal friction [1,4]. Cartilage degeneration is associated with chondrocyte senescence, cellular oxidative stress, and cytokine production, all of which elicit a compounded detrimental effect that can ultimately lead to the development of osteoarthritis (OA) [5]. One of the key interventions to help prevent cartilage degeneration is reducing the constant load on joints and promoting their lubrication, two conditions that are usually not met in people that follow a sedentary lifestyle [6]. Mechanical overload in cartilages induces the up-regulation of degrading enzymes such as metalloproteinase-13, which contribute to the degradation of the tissue matrix [7], along with the reduced release of transforming growth factors, whose stimulatory activities in chondrocytes promote the production of collagen type 2 and proteoglycans to facilitate tissue repair [8,9].

In healthy joints, the cartilage has some ability to adapt to different types of mechanical stimulation [10], like dynamic compressive forces and shear stresses, both of which induce the activation of molecular signaling pathways that allow the growth of its epiphyseal portion [11]. Indeed, moderate constant and compressive mechanical forces are necessary to increase the synthesis and concentrations of proteoglycans, glycosaminoglycans, and the other components of the cartilage matrix [3], and also to regulate the rate of the turnover of cartilage molecules within the joint. Therefore, physical movement is considered to be a non-harmful and fundamental activity for maintaining cartilage health and cellular homeostasis [12,13], whilst also providing protection against the development of OA [14]. Aerobic, endurance, and aquatic sports [15], at different intensities, seem to elicit positive effects on the maintenance of cartilage integrity, both in terms of thickness and by increasing the volume of the chondrocytes’ nuclear density per unit area [16]. In the absence of underlying pathological conditions of the joint, cartilages undergo transient positive adaptive changes upon stimulation via physical activity; however, these effects are sustained only after repeated sessions [17], suggesting that the routine practice of sports activities may indeed promote cartilage health [1,18,19]. Physical exercise is also beneficial for people at risk of developing OA or with overt pathology, considering the positive stimulation of physiological cartilage turnover and its ability to prevent the increase in the release of inflammatory molecules associated with the disease [20]. Moreover, physical activity can also promote pain relief in OA patients [21], and it is used as a non-pharmacological co-treatment that can limit the need for subsequent pharmacological interventions with opioids, non-opioid analgesics, and/or non-steroidal anti-inflammatory drugs—the gold-standards for pain management in OA [22]. However, before prescribing physical exercise to individuals, it is vital to establish the correct regime and intensity of exercise to achieve the most benefits for cartilage health and to render the intervention as effective and safe as possible, as under- or over-stimulation of joints can lead to the lack of therapeutic effect or even damage [23]. For this purpose, standard operating procedures could be adopted to guide the correct implementation of physical activity and improve the quality of the intervention [24]. 

In the literature, there are numerous biomarkers used to evaluate the different aspects of cartilage/joint health, such as bone metabolism, molecules released within the synovium, and inflammation biomarkers [25,26]. However, the myriad of markers discovered so far, whilst helpful for understanding certain aspects of cartilage pathophysiology, paradoxically have also contributed to diverting rather than guiding physiatrists and other therapists in defining qualitatively and quantitatively the best interventional approach. A reason could be that many of these putative cartilage biomarkers are not as specific indicators of cartilage health as they should be [27]. Another aspect to bear in mind is that, pre-clinical and/or in vitro studies assessing knee joint cartilage deformation may not have the expected translational value in humans [28], pinpointing the need for further in vivo experimentation in humans [16]. 

Many studies have been conducted over the years on OA biomarkers, identifying many options for evaluating its progression and severity; however, the literature has been widely heterogeneous when physical activity is an included variable. Considering the importance of regular physical activity for articular cartilage health, the broad heterogeneity of findings in published studies [29], and the importance of personalized protocols and treatments (especially for people with OA) [30], and also taking into account that it is fundamental to better understand the role of the intervention on cartilage health through an appropriate and comparable evaluation, it is consequently important to standardize the evaluation methods by adopting the same biomarkers, and for this reason, standard operating procedures are required [24]. Consequently, this review aims to narrow the evaluation of relevant articular cartilage biomarkers, with emphasis on those markers that can draw some benefits from physical activity, and eventually propose a standard operating procedure. These analyses may aid in the development of tailored approaches to improving cartilage health and instigate further and more streamlined research in humans.

## 2. Methods

This review partially followed the preferred reporting for systematic reviews and meta-analyses for scoping reviews (PRISMA-ScR) checklist and explanation [31]. The protocol of the manuscript has not been previously published.

### 2.1. Search Strategy

The search was performed on Pubmed, Web of Science, and Scopus until 21 September 2022, and all articles published were considered regardless of the date of publication. Keywords for the subject (articular cartilage, fibrocartilage, and gristle), intervention (exercise, physical activity, fitness, movement, and sport), and outcomes (collagens, chondron, matrix, collagen, proteoglycan, and synoviocyte) were connected with the Boolean operators AND and OR. 

The following string was adopted in the three databases searched:

(“articular cartilage” OR fibrocartilage OR gristle) AND (exercise OR “physical activity” OR fitness OR movement OR sport) AND (collagens OR chondron OR matrix OR collagen OR proteoglycan OR synoviocyte).

### 2.2. Eligibility Criteria

The population investigated had to be composed of active or sportive individuals, and the intervention and the terms of comparison adopted had no restrictions. The outcomes considered were only biomarkers classified as “burden of disease” [32]. Regarding the study design, only original, peer-reviewed, and English-language manuscripts were included independently of the origin country.

### 2.3. Screening Process

Two investigators performed the collection and screening against the eligibility criteria. The screening was performed by title, abstract, and full text. Manuscripts were retrieved also by checking the references of the included papers.

All the collected papers underwent narrative analyses, and relevant biomarkers were then presented and discussed.

## 3. Results

A total of 4342 (PubMed: 1874, Scopus: 1077, Web of Science: 1391) articles were found in the three databases searched. After duplicate removal, a total of 2685 articles were screened against the eligibility criteria. Following the exclusion of papers about animals or direct interventions on the cartilage (i.e., implantation, treatment), about treatments other than physical or sports activities (i.e., diet, medical treatment), or in injured people (i.e., cruciate reconstruction, muscle injuries), a final number of 24 studies were included. The flow diagram can be found in the Supplementary Materials.

### 3.1. Narrative Synthesis of the Results

The main burden of disease biomarkers identified in the scoping review were the cartilage oligomeric matrix protein (COMP), detected in 16 studies, the carboxy-terminal telopeptide (CTX-II) in 5 studies, interleukins (ILs) in three studies, the matrix metalloproteinases (MMPs) in 2 studies. Several other biomarkers have been analyzed in different studies: in one study, circulating levels of serum C-reactive protein (CRP) combined with cartilage type II collagen measurement was used as a parameter of general inflammatory reaction and cartilage metabolism in marathon runners [33]. Similarly, some studies used the sGAG [34,35]. A detailed description of the main burden of disease biomarkers is in the paragraphs below.

#### 3.1.1. Cartilage Oligomeric Matrix Protein: COMP

A total of 16 studies investigated the sCOMP. Most of the studies investigated the articular biomarker after a running intervention (seven times), followed by walking (four times), and general (three times) exercises. Drop landing and soccer interventions were studied two times, while resistance exercises, jumping exercises, cycling, volleyball, basketball, weightlifting, and swimming one time.

COMP is a 535-kDa non-collagen extracellular matrix component of the thrombospondin family proteins and it is primarily found in the articular cartilage, tendons, and synovium [36]. It is a widely adopted non-collagen biomarker for cartilage turnover within the articular cartilage, and most of its structures remodel the vascular walls [37,38]. The biomarker can be extracted by a serum (sCOMP) or in the synovial fluid, and both are investigated to predict the OA progression in the knee [39,40] even if synovial fluid COMP seems to have a better predictive value than serum COMP [36]. sCOMP is measured in ng/mL and is usually collected immediately after, at 30 min, 1, 2, or 3 h post-physical exercise [41]. COMP is associated with different diseases and it is a well-known biomarker for pathologies that elicit cartilage destruction such as osteoarthritis, rheumatoid arthritis, intervertebral disc degeneration, and psoriatic arthritis [42].

The level of sCOMP increases immediately after physical activity and transiently elevated levels have been detected in athletes from different disciplines such as running [28,41,43,44,45,46,47], cycling [44], soccer [48,49], walking [37,50,51,52], resistance training [50], physical activity [53], and drop landing (box high 73 cm) [28,37] or jumping exercises [47]. sCOMP levels seem to slowly reduce until returning to baseline within 30 min from exposure to a physical challenge [44]. The effect of different sports on detectable sCOMP levels is not significant when comparing elite volleyball, soccer, basketball, and weightlifting [54], or between cyclists and runners [44]. In a study conducted in a cohort of female soccer players after a full training season, no significant changes in sCOMP levels were detected [48]. After 3 h of activity, the levels of sCOMP are lower (about 16%) than the values detected immediately after the end of the effort [41], and its decrease continues until the 5.5 h sample [52]. sCOMP concentration increases again at 5.5 h after the end of physical activity, indicating a metabolic delay for sCOMP in the range of 5 h to 6 h [52]. When the activity is prolonged over a long period, for instance, in an ultramarathon, the sCOMP concentration levels remain stable after the first adaptation [46]. The concentration of sCOMP seems to be dependent on the impact and the loading of the articulation [28]. Indeed, a study increased the knee joint load, and the investigators detected higher COMP levels with higher knee joint moments [43]. Another study emphasized that the COMP could be released from cartilage: COMP degradation and clearance vary depending on joint loading characteristics [55], and it was found that the impact of running (and perhaps other types of physical activities) promotes the conversion of COMP into sCOMP and its translocation from the joint cavity to the bloodstream [45]. This could be explained by a study that suggests how sCOMP levels are likely to be associated with muscular co-activation [56]. The response to exercise seems not to differ between genders, but generally, there is a trend toward higher COMP concentrations in males than in females at all time points [50]. In females, the thinner anterior femoral cartilage was associated with higher resting sCOMP concentrations [37].

It was found that in people with OA, a combination of aerobic, muscle strengthening, and flexibility exercises can increase sCOMP concentrations [57]. Furthermore, acute exercise has a COMP-lowering effect within the knee joint [58] and there is a beneficial effect of strengthening exercises with a reduction of the circulating sCOMP levels [59].

#### 3.1.2. Matrix Metalloproteinases (MMPs)

MMPs were studied in only two studies, one on ultramarathons [46] and one on running and jumping [47]. MMPs are proteolytic enzymes belonging to the family of the adamalysins, serralysisn, and actacins, with a role in the degradation of the proteins of the extracellular matrix, and play a crucial role in cartilage destruction [36]. MMPs are considered useful biomarkers in the diagnosis and prognosis of different types of OA [36]. Serum concentrations MMP-1, MMP-3, and MMP-9, acutely adapt to mechanical loading [47], with rapid increases at the beginning of physical activity followed by a fairly rapid stabilization thereafter [46]; moreover, MMP-2, MMP-3, and MMP-9 are highly expressed in patients afflicted by arthritis [60]. In relation to MMP-1, at least one study failed to detect any changes in its concentration throughout an ultramarathon, suggesting that levels of this metalloprotein may not be affected during strenuous exercise [46]. Another MMP is matrilysin, also known as MMP-7, which is abundantly expressed in the human cartilage and is significantly elevated in people with OA compared to healthy controls, and was correlated with high levels of IL-15 [61]. MMP-8 is a collagen-cleaving enzyme that plays a role in cartilage degradation, driving the osteoclast-mediated release of glycoaminoglycan (GAG) [62]. Stromelysin 2 (aka MMP-10), which is similar to MMP-3, is involved in OA progression, due to its ability to activate pro-collagenases and play an active role in the proteolytic cleavage of the cartilage extracellular matrix [63]. The matrix metalloproteinase 12, MMP-12, was associated with cartilage destruction in arthritis [64], considering that it can degrade different extracellular matrix components, including elastin, collagen IV, fibronectin, laminin, gelatin, vitronectin, entactin, fibrinogen, plasminogen, and chondroitin sulfate [63,65,66]. MMP-13 has a central role in OA due to its role in the degradation of type II collagen, the most abundant type of collagen found in the articular cartilage [67]. MMP-13 degrades larger collagen fibrils [68] and a recent study evidenced its potential usefulness as a biomarker for knee OA [69].

#### 3.1.3. Carboxy-Terminal Telopeptide (CTX-II)

A total of five studies adopted CTX-II: general exercise and swimming were proposed two times while resistance training, running, cycling, and futsal only one time. CTX is a fragment of the telopeptide of collagen, and they both derive from the bone, type I (CTX-I), and the cartilage, type II (CTX-II). While CTX-I is a serum biomarker of bone turnover, CTX-II is a biomarker of articular cartilage degradation [36]. It is often evaluated in its urinary form (i.e. uCTX-II) and it is considered as reliable as serum CTX-II as a biomarker of OA [36]. A reduction in circulating levels of CTX-II was detected in patients with OA that practiced physical exercise [70] and strengthening exercises [59]. Likewise, higher uCTX-II has been detected after exercise training [57], portraying it as a negative indicator of cartilage health status. CTX-II was found to be significantly higher in runners than in crew, swimmers, or controls [71], suggesting that running is an activity that could increase the risk of collagen degradation and consequently articular cartilage degradation. Similarly, CTX-II values were higher in futsal players (a variant of football played on a hard court) than in swimmers [72]. It was also found that people with OA exhibit high levels of uCTX-II compared to healthy controls and these levels correlate with the Kellgren–Lawrence grade of OA [73], which was also confirmed by a recent meta-analysis that synthetized 13 studies on uCTX-II, knee OA, and Kellegrad–Lawrance OA severity [74]. Surprisingly, from a randomized cross-over trial in patients with OA, it emerged that cycling induces a higher concentration of cartilage degradation biomarkers compared to running [75]. Moreover, it seems that traumatic injuries partake in cartilage deterioration [76].

#### 3.1.4. Interleukins (ILs)

ILs were proposed in five studies, endurance activities in three studies, while two studies proposed an exercise routine. Interleukins are well known inflammatory markers. IL-10 is a cytokine with chondroprotective anti-inflammatory and anti-apoptotic properties [77]. Considering that apoptosis-related factors are associated with cartilage degeneration [78], it is fundamental to highlight the positive effects of IL-10 seen in people with OA, as the interleukin interferes with cell death, joint inflammation, and the matrix degenerative process [68]. IL-10 is produced by articular chondrocytes and synovial fibroblasts [79] and seems to elicit beneficial effects on the activity of articular chondrocytes [80]. The practice of physical activity, especially that involving repeated loading of the articulation (i.e. endurance exercises), increases IL-10 at both intra-articular and pre-synovial levels in humans with knee OA [70,81]; moreover, IL-10 also stimulates the synthesis of CTX-II [82]. Another interleukin, IL-1, has been identified as the principal mediator of cartilage degradation associated with OA, and its activity is regulated by the IL-1 receptor antagonist [83]. IL-4, similarly to IL-10, has chondroprotective effects, as it influences proteoglycan metabolism and inhibits the activity of several MMPs, preventing the apoptosis of chondrocytes [82]. In addition, IL-1β, IL-6, and IL-8 displayed significant increases over time during the practice of physical activity [70,81]; in particular, IL-6 is considered a key factor related to cartilage degeneration and pain [84]. IL-15 concentration decreased after running [45].

## 4. Discussion

Different molecular burdens of disease biomarkers have been investigated to address the effects (positive or negative) of physical activity on the articular cartilage and the most commonly adopted ones are COMP, certain MMPs, CTX-II, and a number of ILs. All of them display different trends in response to exertion and may be useful for determining various aspects of cartilage health. Amongst these, the most frequently adopted is COMP. Considering the findings, a standard operating procedure could consist of taking sCOMP samples always at the same time of day. It is important to specify the hours and the time elapsed since the last training. It is fundamental to describe the participants’ characteristics, not only in terms of disease but also their diet and working experience. A final aspect to consider is the need to deeply describe the activity practiced in terms of frequency, time, type, and volume. It is also important to collect data about their physical background. Only if all this information is provided, will it be possible to compare the studies in the future and improve the quality of the research in this field. A synthesis of the proposed standard operating procedure is presented in Table 1. Below, the rationale behind the standard operating procedure is discussed.

Data from several recent reports [36,85,86] portrayed COMP, MMPs, CTX-I, CTX-II, and IL-1b as valid biomarkers for OA. There is heterogeneity in the published studies on this topic, most likely due to the variable selection of biomarker panels [29]. This critical aspect highlights the importance of establishing: (1) the reliability of the chosen biomarkers, as well as (2) defining the most suitable panel of biomarkers for OA and cartilage health in general. Such a systematic approach is perhaps what is needed to allow standardization of these biochemical parameters across studies, thereby improving comparability and the interpretation of results. From the literature, it emerges that sCOMP is currently appreciated as one of the most frequently interrogated markers of cartilage health [25]. Additionally, levels of sCOMP remain stable both within minutes (interday) and 1–2 days post-exertion (intraday), demonstrating both strong interday and intraday reliability [87]. Nonetheless, sCOMP levels fluctuate following circadian rhythmicity patterns, like CTX-II [88], so it is recommended that sampling is always performed at the same time of the day (best in the morning), and in patients that follow a stable dietary regime [88]. Furthermore, sCOMP appears to be better for the detection of knee OA, while CTX-II seems more specific for the detection of hip OA [36]. Combined, sCOMP and CTX-II exhibit robust features to be included in the aforementioned biomarker panel, at least to complement imaging findings and/or clinical evidence in subjects afflicted by OA. 

Physical activity is fundamental to maintaining cartilage health and homeostasis [89]. Indeed, the stimulation of the cartilaginous superficial zone caused by shear stress and hydrostatic pressure can increase CTX-II synthesis [90]. As mentioned, joint loading also promotes cartilage metabolism in the attempts at reducing cartilage degradation during and after prolonged exercise. This is reflected by the higher sCOMP levels in these individuals, long-lasting increases in which may elicit beneficial effects lasting for several days [25]. Exercise is considered a metabolic modulator and the metabolic changes it induces may be partially responsible for an improved cartilage health outcome. However, the mechanisms by which exercise prevents disease occurrence and improves physical recovery in chronic joint illnesses are still poorly understood. An increasing body of evidence suggests that the gut microbiome may be implicated in this process [91]. It is also necessary to consider that one factor that also contributes to cartilage degeneration is chondrocyte senescence, articular oxidative stress, and the production of pro-inflammatory cytokines [5]. The positive effects of physical activity on cartilage are determined both by the intensity and typology of the activity. Clearly, a great body of evidence has also demonstrated how inappropriate training regimes and/or supraphysiological movements can elicit deleterious effects on the articular cartilage [6,92,93,94,95]. By contrast, it seems that regular cyclic loading enhances proteoglycans synthesis (this improves cartilage stiffness), while there are fewer effects on the articular cartilage collagen fibril network [4]. If the compression is continuous, it diminishes proteoglycans synthesis, increasing the risk of tissue damage [4]. This highlights the need to understand the responses of the molecular biomarkers to the intensity and the typology of physical activity. In this respect, it should be interesting to quantify the responses in healthy people and patients with OA according to the severity of the disease. 

This study aims to provide an overview of the principal biomarkers adopted in the evaluation of the articular cartilage in healthy people and in patients with OA. The ultimate goal is to provide researchers but also experts in the sector with an instrument to better understand and standardize the use of these biomarkers in future studies that evaluate cartilage and the effects of physical activity. In this way, it will be possible to compare the data and to try to create guidelines to establish the correct frequency, intensity, duration, and type of activity for achieving the best outcomes for cartilage health in the healthy and diseased populations. This review could represent a valid tool for researchers to acknowledge the current state of the field of cartilage biomarkers mainly employed in studies on cartilage and physical activity.

## 5. Conclusions

There is a huge variety of molecular biomarkers currently under investigation to reveal the conditions of articular cartilages, either after simple mechanical stimulation and/or during/after physical activity. The most frequently adopted markers are sCOMP, CTX-II, Ils, and MMPs, and their use in combination seems to provide sufficient data to determine whether crucial protective and regenerative mechanisms are activated within weight-bearing joints by physical exertion both in healthy people and in patients with OA. A standard operating procedure for a burden of disease molecular biomarker evaluation of the cartilage during physical activity practice has been proposed.

## Figures and Tables

**Table 1:** Standard operating procedure for the burden of disease biomarkers to be used for articular cartilage assessment during physical activity practice.

	Main Indication	Secondary Indication
Burden of disease biomarkers	serum cartilage oligomeric matrix protein (sCOMP)	sCOMP seems better for knee OA; CTX-II seems more specific for the detection of hip OA
Primary point	Time of the sample	Time elapsed since the training
Secondary point	Description of the sample	Description of the treatment
Tertiary point	Description of the training	Description of the background

Carboxy-terminal telopeptide: CTX-II; osteoarthritis: OA.

## Data Availability

Detailed information of the screening process and article selection are available contacting the first Autor.

## Supplementary Materials

The supporting information can be downloaded at: https://www.mdpi.com/article/10.3390/ijms24043662/s1. Reference [96] is cited in the supplementary materials.
