# Supplementary material for: Molecular Assessment of Healthy Pathological Articular Cartilages in Physically Active People: A Scoping Review
Source: Int J Mol Sci. 2023 Feb 11;24(4):3662. doi: 10.3390/ijms24043662 (PMC9963910; doi:10.3390/ijms24043662)
Supplement: Supplementary file 1 [file ijms-24-03662-s001.zip › ijms-2161017-supplementary.pdf]

PRISMA 2020 flow diagram for new systematic reviews which included searches of databases and registers only

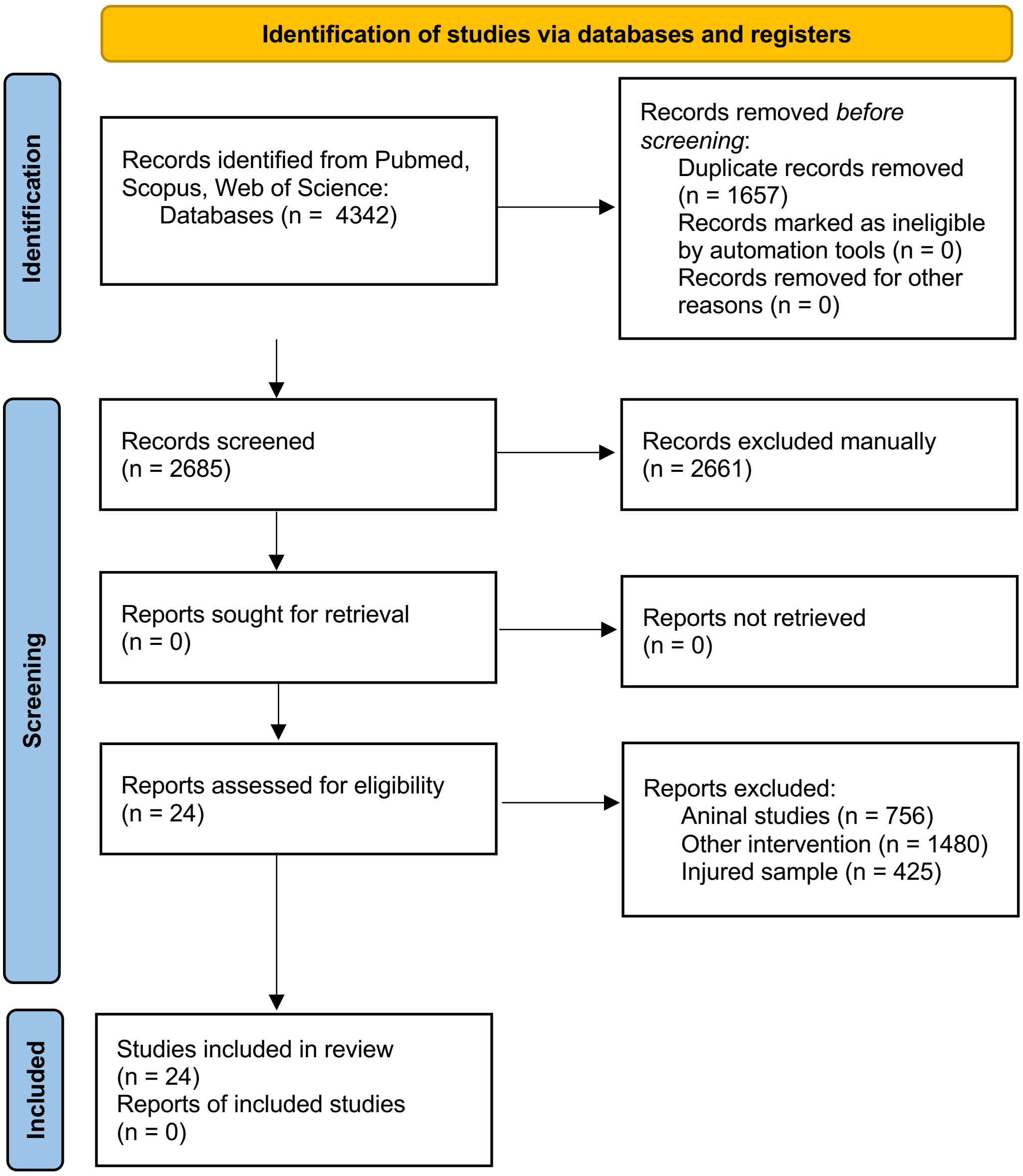

From: Page MJ, McKenzie JE, Bossuyt PM, Boutron I, Hoffmann TC, Mulrow CD, et al. The PRISMA 2020 statement: an updated guideline for reporting systematic reviews. BMJ 2021;372:n71. doi: 10.1136/bmj.n71

For more information, visit: http://www.prisma-statement.org/
